# Editorial: Chordoma: advances in biology and clinical management

**DOI:** 10.3389/fonc.2023.1175683

**Published:** 2023-05-05

**Authors:** Paul Gardner, Jiwei Bai, Sebastien Froelich, Xiaohong Rose Yang, Cheng Yang

**Affiliations:** ^1^ Department of Neurological Surgery, University of Pittsburgh Medical Center, Pittsburgh, PA, United States; ^2^ Department of Neurosurgery, Beijing Tiantan Hospital, Capital Medical University, Beijing, China; ^3^ Department of Neurological Surgery, Assistance Publique Hopitaux De Paris, Paris, France; ^4^ Division of Cancer Epidemiology and Genetics, National Cancer Institute, National Institutes of Health (NIH), Bethesda, MD, United States; ^5^ Department of Orthopedics, Shanghai General Hospital, Shanghai, China

**Keywords:** chordoma, radiomics, biomarkers, tumor microenvironment, proton radiotherapy

## Perspective

Chordoma is a rare bony tumor, occurring across the neuraxis, most commonly in the sacrum or skull base. Generally considered to be a malignant tumor, new evidence has shown that it can have a wide range of clinical behavior (1). This range reflects the same kind of variable molecular underpinnings that impact prognosis of every tumor type, the study of which can help to unlock the drivers of oncogenesis with the hope of defining the key targets for treatment. Current advances in chordoma treatment have arisen from the development of new surgical techniques, application of logical immuno- and chemotherapy trials, refinement of high-dose radiation techniques and individual targeted therapies.

Much of the initial work and motivation which initiated the scientific creativity and surprisingly rapid progress in the understanding of chordoma came from the efforts of the Chordoma Foundation (2). An international patient and scientific community, this foundation has brought together and supported a network of physicians and researchers in an institutionally agnostic setting, encouraging rapid and free sharing of results, even before publication. This has allowed a large number of clinicians and researchers to learn rapidly from each other and drive understanding and progress in a logical and cohesive fashion.

Tumor research in neurosurgery has largely focused on glioblastoma (GBM). However, given the complexity and rapid transformation of this disease, these efforts have gone largely unrewarded with respect to impactful treatments. However, applying the same rigorous study and techniques to other tumors such as meningioma and chordoma have, at a minimum, led to a dramatic improvement in understanding of the genetic range that drives behavior and prognosis despite similar histology. This understanding is the first step in personalized treatment for patients and begins to unlock the changes within these tumors which may provide new and effective treatment options. It is likely that applying similar efforts to chordoma, as have been applied to GBM, will yield far greater success as it has in head and neck surgery. It is with this hope that this Research Topic was assembled with each article providing a small step along this path. Covering topics from radiomics to circulating DNA and new drug targets, this article collection will hopefully serve as part of the foundation upon which future successful chordoma research efforts will be built.

## Article summary

The articles cover every aspect of chordoma, from predicting prognosis to treatment options to molecular analysis and personalized medicine. For the first time, radiomics have been applied to chordoma. The article by Zhai et al. shows that radiomics can be used to correlate chordoma prognostic categories. This would potentially allow preoperative prediction of behavior to allow tailored surgery. It could also be useful in settings where advanced molecular techniques are not available or when the tumor sample is too small to fully classify the tumor. Adding to that, further work on prognostic biomarkers in a systematic review by Rubino et al. help identify which biomarkers may provide the best surrogate for prognosis. Furthermore, Xiong et al. describe PALB2 (Partner and Localizer of BRCA2) as a novel prognostic factor, solidifying the concept of grades of chordoma and allowing for refinement of future radiomics. New work revealing the potential role of the tumor microenvironment (TME) in chordoma progression identified a correlation between systemic inflammatory score and adverse outcome (Li et al.). On the opposite end of the spectrum, Lopez et al. evaluated the immune microenvironment using immunofluorescence techniques and revealed spatial immune composition in chordoma TME. As with any tumor, understanding potential immunotherapies could go a long way toward controlling disease.

This Research Topic also evaluates treatment options, from a comprehensive review of new options to a systematic review of proton radiotherapy for chordoma and chondrosarcoma, to a study on the role of Gamma Knife Radiosurgery. Seeking to ease the impact of surgery, Fu et al. looked at factors associated with postoperative airway retention in skull base chordoma. Research hotspots are evaluated using bibliometric criteria and other articles present novel concepts for treatment or monitoring and diagnosing disease. Passeri et al. show their work using xenograft models for preclinical testing of therapeutic options. Freed et al. evaluate drug repurposing *via* target discovery and Zhao et al. review techniques for developing new therapeutics. Finally, circulating DNA in chordoma shows significant promise to allow detection of recurrence or even confirmation of diagnosis at initial presentation without biopsy.

As editors of this Research Topic, we hope that it will demonstrate the progress made in the understanding and treatment of this rare tumor. We have all seen the devastation chordoma can cause once uncontrolled; many of us have dealt with the morbidity of our current treatment strategies. Over time, this creates a strong desire for better treatment, to improve our patients’ lives and clarify the role of our treatments in order to limit their impact. This desire underlies all of the work presented herein and hopefully helps to slowly achieve these mutually inclusive goals.

## Author contributions

PG made substantial contributions to the design of the work and drafted and revised it critically for important intellectual content. JB, SF, XRY and CY provided approval for publication of the content. PG agrees to be accountable for all aspects of the work in ensuring that questions related to the accuracy or integrity of any part of the work are appropriately investigated and resolved.

## References

[1] Zenonos GA Fernandez-Miranda JC Mukherjee D Chang YF Panayidou K Snyderman CH. Prospective validation of a molecular prognostication panel for clival chordoma. J Neurosurg (2019) 130(5):1528–37. doi: 10.3171/2018.3.JNS172321 29905508

[2] Available at: https://www.chordomafoundation.org.

